# Wolf–Dog–Human: Companionship Based on Common Social Tools

**DOI:** 10.3390/ani13172729

**Published:** 2023-08-28

**Authors:** Kurt Kotrschal

**Affiliations:** Department of Behavioral & Cognitive Biology, University of Vienna, 1030 Wien, Austria; kurt.kotrschal@univie.ac.at; Tel.: +43-(0)664-8175120

**Keywords:** dog welfare, brain evolution, domestication, human–animal relationships

## Abstract

**Simple Summary:**

A major factor in dog welfare is a good relationship with their humans. In turn, living with a dog supports the wellbeing and even the mental and physical health of their human masters. In fact, the Palaeolithic partnership between humans and wolves was social and cooperative from its beginning; living with humans selected for tame wolves and thereby turned them into dogs, fine-tuning the initial social match even more. Why humans can be social with other animals at all may be explained via a common “social toolbox”—a social brain and physiology—shared between humans and other animals because of both a common phylogeny and parallel evolution. Such “social kinship” between humans and other animals makes it possible to conclude that satisfying the social needs of dogs by providing cooperative and empathic human leadership is crucial for their welfare, and that anthropomorphising dogs on the basis of informed human empathy is not as negative as it may sound; it seems rather that it is an adequate basis for a good partnership for mutual wellbeing.

**Abstract:**

Wolves, dogs and humans share extremely social and cooperative minds. These similarities are rooted in phylogenetic homology and in the convergence of neuronal and physiological mechanisms, particularly the brain, in the functioning and communication of basic affects and in the mechanisms of stress and calming. The domesticated wolves called dogs are particularly close companion animals. Both Palaeolithic humans and wolves were hypercursorial hunters, cooperating in complex and prosocial ways within their clans with respect to hunting, raising offspring, and defending against conspecific and heterospecific competitors and predators. These eco-social parallels have shaped the development of similar social mindsets in wolves and humans. Over the millennia of domestication, this social match was fine-tuned, resulting in the socio-cognitive specialists humans and dogs, possessing amazingly similar social brains and minds. Therefore, it can be concluded that the quality of their relationships with their human masters is a major factor in the wellbeing, welfare and even health of dogs, as well as in the wellbeing of their human partners. Based on their strikingly similar social brains and physiologies, it can be further concluded that anthropomorphically applying human empathy to dogs in an educated manner may not be as inappropriate as previously thought.

## 1. Introduction

It seems natural that humans often live in more or less personalised relationships with other animals. This is of course true for companion animals (pets) and, to some degree, also for livestock—at least until the onset of industrialised meat production. As humans have always lived with pets [1], this lifestyle probably qualifies as a “human universal” [2,3]. In fact, no other species engages in pet keeping in the same way as *Homo sapiens*. However, it is still astonishing that with increasing global urbanisation more, rather than less, cats and dogs are being kept [4]. Why do people want to live with pets, how are they able to establish individualised relationships, including social partnership, and what does that mean for the wellbeing and welfare of the companion animals, as well as of their humans?

The generality of this phenomenon suggests that pet keeping is rooted in a specifically human nature and mind [5]. Erich Fromm [6] and Edward Wilson [7,8] termed this specifically human interest in nature and animals “Biophilia”. The urge to keep and live with pets is probably part of a typical feature of the human mind; this is supported by findings that babies independent of cultural background are born with a strong interest in animals [9,10]. For example, the first vocalisations of toddlers are generally animal related, and media for children is dominated by animal metaphors [11]. These facts are indicative of both the important role of animals in the evolution of modern *Homo sapiens* and their role in the optimal development of children [12].

Virtually all livestock and companion animals are domesticated animals, i.e., genetically adapted to live in a human-dominated environment. The degree to which human–animal relations have changed the face of the earth is exemplified by the fact that today, roughly 95% of the biomass of land-living vertebrates is comprised by humans and their domesticated animals [13]. To an alarming extent, wildlife is prosecuted, and its habitats claimed for human use [14]. An estimated one billion cattle and 1.3 billion sheep graze the world’s pastures; 780 million pigs and 35 billion chickens are fattened towards their early end [15]. Of the estimated 1 billion dogs in the world, approximately 470 million are kept as companion animals, and of the 600 million cats, 220 million [4]. The general claim of modern humane societies is that the wellbeing and welfare of these domesticated animals, including livestock, needs to be ensured by adequate keeping conditions. One of the central factors in this is the social environment, as virtually all domesticated animals are highly to reasonably social (e.g., dogs versus cats). 

Following this short excursion into the ultimate-level spheres of human–animal relations (in the sense of Tinbergen’s four levels, from 1963 [16]), I will in the following focus on the proximate and ontogenetic levels, substantiating why people are not just willing, but indeed, able to relate to other animals and to engage in true social relationships with at least some of them, and why this is relevant for animal welfare. In the light of the long-standing and self-proclaimed position of humans in the West as the “crown of creation”, it is still not self-evident to many why *Homo sapiens* can engage in more than utilitarian relationships with other animals, and vice versa. In the following, I will argue that this is possible because of a common “social toolbox”: due to phylogeny as well as, partially, to parallel evolution, humans share with other animals most of the components and mechanisms of their social brains and physiologies. For example, the brainstem–diencephalic “social network” [17], which commands instinctive socio-sexual behaviour, has virtually remained structurally and functionally unchanged since the Palaeozoic [18]. Other examples of conservatively preserved (i.e., evolutionary homologous) common social tools include the mechanisms of stress management and calming [19,20,21] and not the least the inventory of basic affective systems [22,23] that humans share with other animals. 

The basic elements of the social brain emerged over 600 million years of phylogeny [24,25]. In contrast, a striking example of parallel evolution (i.e., an analogy) would be the functionally identical mammalian prefrontal cortex and the Nidopallium caudolaterale of birds, which are composed of identical neural elements, but are phylogenetically and ontogenetically assembled into very different structures [26]. One of the major selective pressures for this parallel evolution was probably the need to control for appropriate behaviour, particularly in socially complex species. I suggest that such knowledge of genetic and social kinship can support respect for other animals and their wellbeing under human control, including in zoos, and as livestock and companions.

## 2. Generating the Relevant Questions

In the following, this review will focus on a number of different topics: first, the reasons for which humans can be social with other animals; second, the reasons for which a descendant of wolves, the dog, became “man’s best friend”; and finally, what this means for the welfare of dogs as well as of their human partners. 

In search of common principles allowing for between-species social relationships, one should first identify the appropriate candidate mechanisms, based on what is known to control/influence within-species social behaviour. Some of these may be more specific than others. For example, the attachment/caregiving behavioural system [27,28] would be specifically social, whereas the appetitive affective system (the “seeking” system, according to Jaak Panksepp [22,23]) would be general, providing the drive for virtually all domains of behaviour, including the social domain. Of course, ultimately all systems that keep organisms alive contribute to social behaviour. Hence, the following listing of major features and mechanisms involved in social behaviour is neither meant to be exclusively social, nor all encompassing. 

Starting from the top down, at a psychological level, any reasonably complex social behaviour needs a social mindset. A basic feature of social complexity is the ability to maintain long-term individualised dyadic relationships between members of a group. Such relationships need individual recognition and social working memory, i.e., the ability to remember social episodes. It also requires the ability to adjust one’s behaviour to the social context, which requires some awareness of one’s own position in the social web. In addition, it needs mechanisms to inhibit/control affective behavioural impulse and for delaying actions, to allow individual experience to translate into appropriate social behaviour, both within and between species. All this cannot be achieved by stimulus–response mechanisms alone; rather, context-specific behavioural flexibility requires mental representations on the relevant details of the socio-ecological environment, i.e., social episodic memory [29,30].

“Appropriate social behaviour” ultimately (sensu Tinbergen 1963 [16]) means balancing one’s own interests with optimal group functioning (i.e., acquiring the necessary resources, defending against competing groups and predators). This requires some basic empathy for judging when one’s own claims and ambitions reach the embarrassment limits of others. In essence, “appropriate social behaviour” is also about the regulation of distance and closeness. Within species-specific limits, proximity seeking will depend on the quality of particular long-term dyadic relationships, determining the potentials of social support in the different behavioural domains, including affective calming [31]. In fact, in socially complex animals—including humans—appropriate social support is a key factor for individual welfare; social success is an important element of mate quality, and hence affects evolutionary fitness [32]. In a way, this is the extreme opposite to systems in which mating occurs in leks, where “social contact” between sexes is reduced to sperm transfer.

## 3. Humans as a Reference?

Because, ultimately, all research of biological principles is self-referential [33] and because of the fact that humans evolved in the same way as every other species, in more or less close phylogenetic relationships to the other DNA systems we call “species”, it seems appropriate to use the human condition as a reference and anchor for understanding of other animals.

Within the vertebrates, humans have clearly evolved into the top socio-cognitive specialists [5,24], as reflected by their having the relatively largest (fore)brains, containing the most neurons [34]. However, brainpower comes at the cost of evolutionary constraints in other functional domains, such as digestion and reproduction [35,36]. Additionally, specialisation generally constrains future adaptation to disturbance and environmental perturbation. In alignment with human nature [5] and socio-cognitive specialisation, optimal individual development and performance requires a certain set of environmental conditions. Above all, bodily, emotional, social, and cognitive development supporting mental resilience in adulthood requires reliable and sensitive caregiving early on [27,28], as well as growing up in contact with nature and animals [37]. Growing up in the good company of animals supports the healthy development of children and may have positive effects on the wellbeing and health of adults [38]. In fact, the growing numbers of companion animals, particularly cats and dogs, kept today in increasingly urbanised societies hint at the social support function of companion animals in a time of multiple global crises. Hence, the available evidence indicates [39] that *Homo sapiens* has seemingly adapted to live with other animals and to profit from it.

## 4. Human–Animal Companionship: Is It Social Abuse?

However, what about the “other side”? Do humans socially abuse and exploit their companion animals? In fact, it can be regarded as a human universal to anthropomorphise other animals [40] in a *scala naturae* way: the phylogenetically closer or the more familiar other animal species are, the more people—relatively independently of culture [41]—attribute human-like mental abilities of thinking, feeling and intentionality to them. From an animal welfare perspective, this is generally assumed to be inappropriate, as other animals have species-specific and individual needs that are different from those of humans. However, how different are they? Is it appropriate to keep animals as pets or in zoos, based on best intentions, i.e., on the basis of human or even humanistic ideas regarding their welfare? Can human empathy be trusted in this respect, or is it vastly inadequate as a basis for caring for other animals, notably dogs? A closer look at the phylogenetic development of human intelligence and mental abilities, and on the common social toolset due to this common evolutionary question may provide relevant perspectives on this. 

To me, as a positivistic scientist, it is still agreeable what Konrad Lorenz insisted on as early as the 1930s—that all mental and behavioural phenomena are caused by the physico-chemical processes in the brain; and I would add here that this is true for all processes in the rest of the body. Hence, for the understanding of all features originating from organismic evolution, including social behaviour, different levels of explanation need to be integrated, including structures and mechanisms, evolutionary functions, ontogeny, and evolutionary history [16]. 

## 5. Vertebrate Brain Evolution as the Basis for a Common Social Mindset

An evolutionary perspective is needed to understand why the brain and other mechanisms can serve as a basis for within-species and between-species social interactions, and also to appreciate the conditions supporting welfare or causing suffering. How did the vertebrate brain evolve, and what were the crucial steps towards mammalian/primate socio-cognitive abilities? The basic answer to this is: through a combination of key innovations [24,25,42] and a very conservative brain evolution. As a rule, ancient neural elements are conservatively kept and put into service in new ways when needed. For example, the oxytocin (OT)–arginin–vasopressin (AVP) system has probably existed since the beginnings of vertebrate brain evolution, some 600 million years ago, when it mainly dealt with homoeostatic regulation. In amniotes, a few hundred million years ago—and a few amino acid substitutions later in the peptide hormones involved—this system increasingly assumed roles in reproduction and social behaviour [43]. However, there are also much more recent adaptations towards a brain fit to handle complex social behaviour, such as the mammalian prefrontal cortex and the analogous Nidopallium caudolaterale in birds [26]. 

**Figure 1:**
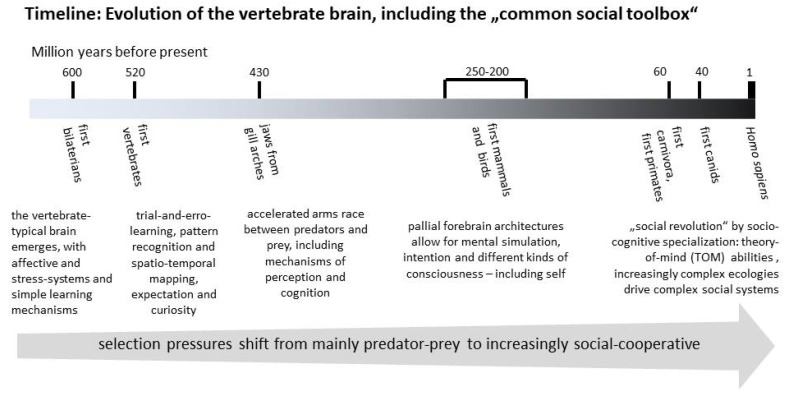
Timeline of the evolution of the vertebrate brain, marking major innovations according to [24,25], driven by selection pressures leading to socio-cognitive specialisation.

The sketch of the evolution of vertebrate brains provided in Figure 1 mainly follows Max Bennett [24,25]. Some 600 million years ago, within the early eumetazoans, a bilaterian Bauplan (in contrast to the radial symmetry of the cnidarians) emerged, necessitating sensory systems and integrative nerve cells at their anterior pole. From early on, the rostral neurons assembled into a structural pattern, which already showed the same serial arrangement that still characterises the vertebrate brain today: the ventral brain stem–Diencephalon continuum and the dorsal Rhombencephalon, Mesencephalon and Telencephalon. Such overall structural and functional evolutionary conservatism of the brain in general also appears in its subsystems. This primordial vertebrate brain of the first bilateral animals was already capable of commanding oriented steering and providing appropriate behavioural responses to favourable and unfavourable stimuli. It developed systems for affective evaluation, stress management and association learning, essentially making it possible to expand the reflex-typical here-and-now stimulus–response relationships over longer periods of time, in order to sustainably deal with environmental contexts and conditions. All of these early integration systems still reside in a nearly unchanged manner in the ventral parts of the brains of humans and other vertebrates [24,25]. 

Some 450 million years ago, the first vertebrates developed jaws from gill arches and engaged in a more active, heterotrophic lifestyle than their filtrating and benthic cordate ancestors. This required systems to generate expectation and curiosity, trial-and-error learning, for remembering and recognising patterns and for acquiring and storing spatio-temporal maps—mainly via an associative forebrain that extended the integration of olfactory stimuli into other sensory domains and towards the potential for cross-talk between domains. All of these early vertebrate developments have been retained nearly unmodified in their currently existing descendants. 

With the first mammals, some 220 million years ago, the telencephalic Neocortex developed, composed of the mammal-typical neural columns; these are basically general-input central processing units (CPUs) with a complex standard neural architecture. With these columns comes the ability not only to adequately respond to relevant stimuli in the environment, but to predict/anticipate the immediate future, which in turn provides a substrate for the development of cognitive tools to mentally play through future options. A great advantage of these columnar CPUs is that they can process inputs from any sensory source. If more brainpower is needed, their number can simply be increased. In the “large brained” mammals this has led to a folded Neocortex. If spread out, the human version would occupy the area of a sizeable tabletop. 

In contrast, the bird telencephalic Pallium shows an amorphous, non-columnar architecture; however, this does not mean that the mental tools for coping with the socio-ecological environment in birds are functionally inferior to those in mammals. Birds emerged in direct continuity from dinosaurs, and hence follow different evolutionary trajectories from the therapsid-originated mammals. In most birds, brain size is constrained by the need to fly. Therefore, the potentials of bird brains are based on neural elements homologous to mammals, but the structure of their pallial Telencephalon developed in parallel. Still, the mammalian prefrontal cortex and the analogous Neopallium caudolaterale of birds show a nearly perfect match of functions [26]. Through miniaturisation, birds pack comparable numbers of neurons into their pallial forebrain to mammals. For example, chimpanzees and corvid birds show similar numbers of pallial nerve cells [44], and, not surprisingly, they excel with similarly impressive cognitive potentials [45]. 

This short tour of over 600 million years of brain evolution, stressing important innovations as well as how conservatively they have been retained, already implies why humans share most of their major tools for dealing with the environment with other animals. This supports the idea that the basic needs and welfare demands of species may be more similar than the distinctions between humans and animals made by philosophers in the Aristotelian tradition, including Bertrand Russell [46]. This idea of genetic, structural and, finally, mental kinship, at least among the amniotic animals, is supported in the following by a closer look at the toolbox of available mechanisms, brain and other. 

## 6. The Common Social Toolbox

### 6.1. The “Conservative” Social Brain

One of the major coordination centres for social behaviour is the so-called “vertebrate social behaviour network” [17], consisting of seven nuclear areas, mainly in the Diencephalon and brain stem. It has remained essentially unchanged in structure, neurochemistry and functionality for 450 million years. This means that modern vertebrates such as humans and their companion animals share this basic central social system with sturgeons and newts. In fact, this is the evolutionarily most conservatively kept part of the overall conservative vertebrate brain [18]. However, this does not mean that humans would share their socio-sexual instinctivity [5] with newts—but the general principles remain the same. What has been adjusted over the course of evolution is, to some degree, the systemic communication and the effector systems [47]. 

Remaining relatively unchanged are also the thalamic/striatal systems of forebrain affectivity; the ancient antagonistic motivation system for approach and avoidance differentiated into more complex affectivity over phylogeny [48], particularly with the emergence of social complexity. Other relatively ancient systems, such as the caudate nucleus and the Amygdala, are relay structures for (socio)positive and fear/stress contexts, respectively. MRT studies of dog brains have revealed that the activity of the caudate nucleus in awake animals increases upon the same socio-positive stimuli—food or social companions—to in humans [49]. These sub-cortical areas support important components of social communication and social life. For example, the twin behavioural systems of attachment and caregiving [27,28], which are relatively instinct-related even in humans, are essential for building and maintaining long-term valuable relationships—both within and between species [39]. 

A core element of attachment and caregiving is the brain–body-connecting oxytocin system, which supports bonding between mothers and offspring, between romantic partners, and even between humans and their companion animals [39]. As an antagonist to the hypothalamo–pituitary–adrenal stress axis [20], the OT system mediates calming [21] when stimulated in sociopositive contexts; it also supports cooperation and trust within groups [50]. In fact, even petting and looking into each other’s eyes can increase OT release in both owners and their companion dogs [51]. The individual expression of the OT system and related psychology are not just based on hard wiring. In humans, the reliability and sensitivity of care experienced in the first year(s) of life determines the primary social mental representation (Bowlby’s “internal working model”, IWM) which serves as a model for engaging in social relationships with social partners later in life [27]. The optimal IWM “secure attachment” may be modified as an adaptive response of offspring to sub-optimal early caregiving into “avoidant” or even “disorganised” attachment, which come with enhanced risks of emotional and social dysfunctions and with the development of problems with learning, social conduct and mental health [52].

Individual attachment patterns also affect relationships with companion animals [39]. In turn, living in good companionship with animals supports the emotional, social and cognitive development of children, and may help to increase their resilience against mental problems in adulthood [39,53], probably because relationships with companion animals are “essentialised”, being virtually devoid of the cultural complications prone to burden relationships among humans [11]. At least in social mammals, the quality of early caregiving (e.g., for a dog pup) seems to have similar effects on lifelong stress management and social behaviour [54]. 

Due to their common evolutionary history, humans share with other amniotic animals at least eight affective brain systems [22,23]: seeking, rage, fear, lust, care, attachment, panic, and play. The communication of affects and emotions (i.e., the subset of affects that makes it into conscious awareness) is at the core of any social relationship, including between humans and other animals [55,56]; being able to read the other’s affects is necessary for being able to develop empathy (within and between species), which in turn is an essential component for creating animal-welfare-friendly conditions. It is possible to communicate affects even between species, because—due to the general organisation of behaviour [57,58]—not only the basic affective systems, but also the principles of their communication are common evolutionary heritage (see below).

### 6.2. The “Modern” Social Brain

Of course, these relatively old parts of the brain cross-talk with the more modern, increasingly “cognitive” and associative parts, such as the mammalian prefrontal cortex or the analogous bird Nidopallium caudolateral (described above); these are the relay centres for conceptualisation, decision making and conscious thinking. Instinctive mechanisms alone would not allow for complex social life; rather, it is the capability of “inhibition” [59], i.e., the ability to control impulses from the reflexive, stimulus–response behavioural systems, that allows individuals to behave “appropriately” in a social web. These modern components of amniote brains ultimately make it possible to balance one’s own interests with those of the others in a group. They allow the development of social cultures with rules regarding to what others can expect and on one’s own limitations, as well as on what sanctions can be expected if these are violated [60]. In this, we see, for example, a difference in how human-socialised wolves relate to human partners compared to dogs. Whereas wolves are happy to cooperate at the same level and even tend to take the lead [61], dogs, during domestication, developed a much stronger respect of hierarchies and they happily obey rules set by their human masters; this, however, does not justify “dominating” dogs, but rather explains why clear human leadership is one of the most important elements in supporting dog welfare. Here, “leadership” is meant in the broad sense of taking the lead regarding the whats and whens of dyadic action, including teaching and learning [62].

Between genetic and epigenetic heritage and the social and societal conditions during upbringing [63], the human prefrontal cortex, and probably that of other social mammals, develops its own individual way of interacting with the (social) environment; “executive function” (EF [64]) is a useful psychological construct for describing the individual quality of impulse control, of the flexibility to adjust to environmental variation, and of the social episodic memory that is distinct from most other forms of learning and memory [65] to support the individual’s ability to pursue strategies and goals, as well as—ultimately—achieve empathetic and social competence. EF is individually optimised during upbringing through sensitive and reliable caregiving, as well as by growing up in contact with nature and animals [37]. It seems that humans develop with a particular sensitivity to these factors [27,28]. Despite the lack of comparative data on the relationship between the conditions during upbringing and the development of EF, it seems that a similar rule may apply in dogs [66]. In any case, adequate human caregiving for dog pups may be considered a main factor in the lifelong potential for wellbeing of dogs in human company.

However, it is not just the “lower” socio-cognitive mechanisms that humans share with other animals. Millions of dog owners experience—and often over-interpret—the astoundingly sophisticated social intelligence of their animal companions. By and large, this is supported by the results of experimental biology. For example (see [61] for a comprehensive treatment of this topic): dogs have been shown to be able to read human affective expressions [67], they express inequity avoidance (i.e., they show a sense of having been unfairly treated [68]), they preferentially lead those human partners to boxes with toys or food that they have previously experienced as cooperative [69], and they may even lead humans they have previously experienced as non-cooperative to empty boxes [70]. 

Such behaviour rules out that dogs would make their decisions exclusively on the basis of stimulus–response mechanisms, a prejudice which burdens human–animal relations till today. However, dogs that differentiate between humans that they have experienced as being differently cooperative must be capable of forming mental representations as a basis for such decision making, in a similar way to humans. This was, in fact, shown by József Topál et al. [71], with their “do-as-I-do” experiments. Dogs can be trained to imitate the bodily action of humans on command. In addition, they will do so after quite some time delay, such as when the human role model jumps over a hurdle and the imitation command is given minutes later. Additionally, dogs judge whether imitating the human action makes sense. When József, for example, jumped over an imaginary hurdle, a few meters away from a real one, the dog would—after some hesitation—jump over the real hurdle. This means that in the sense of the Darwinian phyletic continuum, dogs use similar or even identical cognitive mechanisms to think and make decisions to humans. 

This also means that dogs necessarily operate on the base of some consciousness to be able to utilise mental representations for flexible decision making. As component of the so-called “domestication syndrome” [72], the forebrains of dogs are approximately 30% smaller in size (relative to body mass) than the forebrains of wolves. In fact, compared with similarly raised and kept wolves, dogs have been shown to be less explorative, less persistent, and less good at imitating and understanding causal relationships, but dogs are more respectful of hierarchies and more interested in, and concentrated on, tasks asked of them by humans [68]. However, there is no evidence that dogs are inferior to wolves in general, or in social intelligence. In fact, a comparison of neurone numbers in the forebrains of different carnivores [73] showed relatively low neurone densities in the large brains of bears, hyenas and lions, but also in domestic cats; the group champions in terms of neurone numbers were raccoons, as well as the domestic dog, with no evidence of a reduction of neurone numbers due to domestication. All of these aspects of dog intelligence suggest that their welfare is supported by an environment doing justice to their socio-cognitive potentials. 

### 6.3. Social Physiology

Fitting with conservative brain evolution, the bodily mechanisms for coping with environmental challenges and variability have also remained very much conserved over phylogeny. This is particularly true for the structure and function of the two major stress systems—the “fast” sympathetic–adreno–medullar (SAM) axis, and the “slower” hypothalamus–pituitary–adrenal (HPA) axis—which ready the body for appropriate action [19,20]. The fast “alarm” response via the SAM is initiated in the brain—with a central role of the Amygdala—and is communicated to the effector organs of the body, adrenal, heart, gut, and others, via the sympathetic nerve, also triggering the release of the aminergic activation hormone adrenalin. In contrast, the “slow” response of the HPA readies the body for coping with a stressor based on four evolutionarily prefabricated strategies: fight, flight, freeze or flirt [74]. These potential behavioural responses are supported by the synthesis and release of the steroid hormone cortisol in the adrenal glands, which itself is triggered by a chain of humoral messengers, from the hypothalamus to the pituitary and from there to the adrenals; as steroids cannot be stored within vesicle membranes, they need to be synthesised on demand—which makes the HPA mechanism comparatively slow; it may take a couple of minutes between perceiving a stressor and the arrival of the systemic cortisol peak. Cortisol is primarily a main metabolic hormone, mediating individual “decisions” to increase blood sugar levels in preparation for behavioural action or to store this energy in the short term as glycogen in the liver or long term in the form of body fat. Hence, stress modulation and stress coping strongly and immediately affect metabolic rates in the same way in humans and their companion animals. 

Evidently, stress modulation is crucially important for the welfare of humans and other animals. This, however, does not mean that total avoidance of stressful stimuli and contexts (i.e., those that upregulate neural and hormonal stress parameters, such as heart rate, blood pressure or adrenaline/cortisol levels), is a desirable strategy towards achieving wellbeing and welfare and maintaining good health; because species and individuals are generally adapted to cope mentally and physiologically with environmental variability/challenge, an integral component of a welfare-friendly environment will include occasional positive stimulation of the stress systems within a range the organism can cope with, combined with an effective means to return to baseline (see below). Hence, mental and physical challenges that humans and dog can cope with, e.g., a game of frisbee, agility, or any kind of team activity, may definitely contribute to the welfare of partners, despite (or because of) these activities temporarily increasing stress parameters. 

The stress systems were originally selected to cope with unfavourable physical conditions and predators. Later in phylogeny they became increasingly important in social behaviour, which is reflected by their substantial modulation in social contexts [31,32]. For example, heart rate increases substantially in resting greylag geese (*Anser anser*) when they see their partner becoming involved in an agonistic interaction, or even when watching a conflict between non-affiliated, but higher-ranking flock members [75]. In fact, in humans and other social animals, stress parameters such as heart rate or systemic cortisol are generally modulated to a greater extent by social context than even by locomotion [32,75]. This indicates both the extreme individual importance of the social context, and the superior role of the stress systems in its regulation [32]. 

Favourable social contexts are a central factor in keeping individual stress modulation within welfare-appropriate limits. In contrast, unfavourable social conditions may push stress responses beyond mentally and physiologically sustainable limits. In fact, chronic stimulation of stress systems, particularly HPA, may have deleterious effects on welfare and health, based on the same mechanisms in humans and other animals. For example, fear-generating social networks, mobbing, etc., may result in chronically high cortisol and blood sugar, causing stress-related type II diabetes, cardiovascular problems, neurone loss in the brain, depression, fear-related mental problems and ultimately, a decreased life span. This suggests that favourable social conditions are most important for the wellbeing and good health of humans and other animals, such as dogs.

Whereas social stress can even result in death, socio-positive relationships buffer against stress via the mechanisms of emotional social support [20]. Socio-positive interactions between social companions, stroking, or even looking into each other’s eyes [51] activate the so-called “calming system” [21], with the peptide hormone oxytocin (OT) at its core. OT is synthesised in the hypothalamic preoptic area, stored in the hypophysis and released in a pulsatile manner, because this hormone is only active in the system for minutes. Mammalian OT is primarily involved in giving birth, in facilitating maternal bonding to their offspring, and in lactation [43]. Secondarily, OT is involved in bonding between romantic partners, as well as in initiating and maintaining the bond between companion animals—notably dogs—and their masters [39,53]. 

Physiologically, OT is an antagonist of cortisol synthesis throughout the entire chain of messengers involved. Thereby, high-frequency OT release due to socio-positive interactions mediates calming, with stress-dampening effects that persist for hours or even days. Mainly within groups and between social partners, OT supports social interest, trustful relationships, and cooperation, buffers against the deleterious effects of stress, and enhances resilience against the potential mental problems associated with chronically high cortisol, including depression, anxiety, etc. [20,21]. Hence, the potential social (over)stimulation of the stress systems may be counterbalanced by another social mechanism, mediating calming via socio-positive interactions between (long-term) partners. This creates a safe basis for exploring and learning, for competitive interactions, and generally in defending one’s own interests. In fact, it is well established that social support from human partners may promote calming in dogs by activating the OT mechanism [20,39,50]; even shelter dogs show cortisol reductions due to social support from humans [76,77].

The OT mechanism is important early on in human development, when sensitive and reliable caregiving is required to allow babies to establish their primary social representation [27]. The calming system is also a central factor in the mutual emotional social support provided by an adequate partnership between companion animals/dogs and their human partners [21,51], and is therefore key for the wellbeing, welfare and, finally, health, of partners. Hence, the potential of one partner to provide social support in resonance with the other is at the very core of mate choice in long-term monogamy and friendship, as well as in companionship with other animals [11,12,31]. 

### 6.4. Common Principles of Behavioural Organisation

Mutual understanding between humans and their animal companions is possible because of the principles of behavioural organisation they share due to their common phylogeny. Konrad Lorenz, Nico Tinbergen and Karl von Frisch were awarded the Nobel Prize for medicine in 1973 for their contributions to explaining animal behaviour, including that of humans [16]. According to their theory, instinctive behaviour, including the communication of affect via mimicry and body language, is based on evolutionary prefabricated, and therefore, quick and easy-to-use behavioural elements called “action patterns” [57,58,76]. These behavioural “apps” reside in “pattern generators”, which are assembled from spinal cord motor neurones, and come with distinct features: for example, the motor patterns for “expressions of emotions” [56] in humans [55,78] and other animals are “innate”, and therefore, they are relatively stereotypical within species; they are variable in intensity, but hardly in shape [79]. 

In contrast to the innateness of instinctive motor patterns, correctly interpreting them in conspecifics or even heterospecifics may well require some “innate” motivation (i.e., specific interest), but the ability to read others’ expressions is not simply “innate”. Rather, it depends heavily on (social) learning during socialisation. This explains why, for example, humans and dogs are able to learn to read and interpret affective expressions in the other species. Although affective mimicry is very different in humans and dogs, the behavioural principles of (instinctive) expression are identical. Above all, the necessity of learning to interpret them creates a window of opportunity for between-species affective communication. 

### 6.5. Anthropomorphising

Understanding the affective expressions of another individual is a necessary condition for being capable of basic empathy, which can be defined as recognising the affects and needs of others and responding to them in a complementary or at least appropriate way. This is not automatically positive, as being able to empathise is also helpful for manipulating others for one’s own benefit. Humans are certainly capable of this, but it has also been shown that dogs can manipulate human partners on the basis of what they know about their cooperativeness and knowledge [69,70].

In any case, empathy is another basic element in social interactions, based on what we know and feel about the other. It is hardly disputable that knowledge about their needs helps to do justice to a partner. This is also true for the relationships between humans and dogs—even from the dog’s side, as the animal learns to judge their human partner and to react appropriately in order to optimise the benefits from and pleasantness of a particular partnership. However, how appropriate is it to intuitively empathise with other animals and to attribute feelings and needs to them on the basis of one’s own experience? Humans anthropomorphise other animals or even relevant objects in their environment in order to relate to them and to grasp and integrate them into their species-specific [78] and individual world view. This human universal is mainly based on social brain mechanisms, which also means that humans relate to the world with their human brains, and therefore, they necessarily anthropomorphise [40]. With animals, this works in a “*scala naturae*” way: The phylogenetically closer an animal species is to humans, and the more familiar people are with them, the more mental abilities (emotional and cognitive)—and of greater complexity—will be attributed to them [41]. Can this typically human (?) way of anthropomorphising [80] other animals lead to animal-welfare-appropriate solutions, or are we to a certain extent doomed to socially abuse even our closest animal companions? The present evidence on the common phylogeny leading to incredible homologies and convergences in the social mechanisms of brain and body suggests that human-typical empathy may, indeed, do justice to other animals—if combined with adequate knowledge.

### 6.6. Dogs Are Special—But How “Unique” Are They?

Based on the common “social toolbox” reviewed above, similar social systems and mindsets have evolved under comparable socio-ecological conditions, even in phylogenetically distant species. Primates and canids diverged from a common ancestor that lived more than 60 million years ago. However, modern *Homo sapiens* and wolves (*Canis lupus*) still show striking similarities in terms of lifestyle and social organisation, as well as substantial context specificity in their social behaviour, depending on the densities of prey and conspecifics and how sedentary/territorial, versus nomadic, group traditions are [5,76].

**Figure 2:**
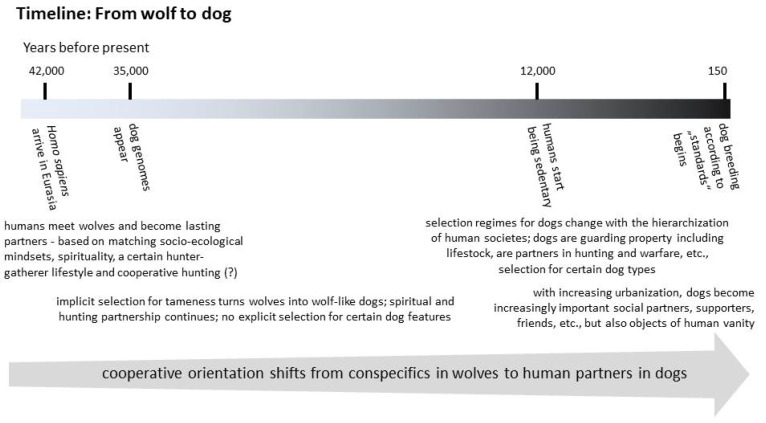
Timeline of major events of the domestication of wolves to dogs [85]. Dog social and cooperative orientation is of wolf heritage, which was adapted during domestication to fit with living with humans [54,61,82,83].

At least some 35,000 years ago, animistic Eurasian hunter-gatherers came into contact with wolves [81,82] (Figure 2)—probably for spiritual reasons. It is very likely that wolf pups were socialised via alloparental human care [83]. However, the common ground for staying together, cooperating over hunting [81,83], and the domestication of wolves and their subsequent evolution towards dogs, primarily via selection for tameness [72,83,84], was a very similar social mindset between these wolves and their Palaeolithic human partners. Both were clan oriented, engaging in complex clan-internal cooperation in relatively flat hierarchies; in fact, dogs require the leadership of their human masters, to the point of it even being a relevant component of their welfare [54,83], whereas human-socialised wolves happily cooperate on the same level with their human partners [61]. Still, more than 50% of the one billion dogs in the world do not live as pets in direct social contact with humans, but as strays, in and around cities, where they scavenge human refuse. These dogs are only able to develop a loose pack organisation, cooperating a lot less than wolves (reviewed in [61]). However, survival and reproductive success of such non-pet dogs still depends greatly on their ability to solicit human tolerance, at least, if not support.

Hence, most of the recent evidence from comparing equally raised and kept wolves and dogs indicates that the latter did not become nice and cooperative through the process of domestication, as suggested by several specific domestication hypotheses (reviewed in [61]). Rather, recent results from comparably raised and kept wolves and dogs indicate that the socio-positive mindset of dogs is of wolf heritage, but has been adapted to serve human needs through the course of domestication. Therefore, dogs are certainly special in being arguably our mentally and socially closest companion animals, but—in light, too, of the basic social toolset shared among amniote vertebrates—they may not be that “unique” in their socio-cognitive features as has been suggested by some mainstream scholars, e.g., [86]. 

There is increasing evidence indicating that living in good relationships with companion dogs supports human wellbeing, health and resilience against mental problems [11,38]. In fact, humans are “biophilic” [6,7,8], and seem to have adapted to live with other animals [11]. Conversely, it seems clear today that dogs living in the good care of and with cooperative and empathic leadership from their human masters enjoy benefits in terms of their welfare, i.e., wellbeing and health. Provided that people live in socio-positive relationships with their dogs, both sides may benefit. However, there are no benefits without costs: the dark side of the human–dog story includes the fact that relationships may go wrong, such as in cases where humans, particularly children, are injured or even killed by dogs [11]. The majority of these accidents do not seem to be predation motivated, but may be a result of social conflicts and misunderstandings. Additionally, dogs can be vectors of potentially severe zoonoses, such as leishmaniasis, leptospirosis, etc., particularly when veterinary care is poor or lacking. Street dogs, in particular, may affect public health. On a global scale, dogs may cause more than 30,000 human casualties per year [87], mainly by transmitting rabies. From a dog’s perspective, symptoms of failed relationships include being euthanised for reasons other than health, or being delivered to a shelter. Still, the positive aspects of the human partnership with dogs prevail. In addition, I would suggest that consciousness regarding the high degree of genetic, social and mental kinship with dogs and other animals—not just as a romantic statement on the unity of life, but on the basis of scientific facts—may support humans’ and animals’ respectfully living together to the betterment of the welfare of both sides. 

## 7. Discussion

In this review, evidence was presented that major neural and physiological social mechanisms are shared among amniotic vertebrates (i.e., birds and mammals, including humans) (Figure 1). This includes the extremely conservative brain social network [17], the stress systems [19], and the oxytocin “calming system” [21]. Such evolutionarily homologous and functionally preserved structures further include the basic affective systems. Of particular importance for socially relating to other individuals within and between species—basically via a largely instinctive behavioural repertoire—are the affective systems of attachment and caregiving [27,28], as well as the motor systems for expressing and communicating affects and emotions [35,77]. However, to allow for complex social behaviour, these basic systems need to be controlled and integrated by evolutionary modern forebrain cognitive centres, such as the mammalian prefrontal cortex and the analogous Nidopallium caudolaterale in birds. It is further suggested that, as a consequence of eco-social adaptation, the social mindsets of humans and wolves are surprisingly similar. 

### 7.1. The Social Environment as a Major Factor in Dog Welfare

The social pack environment is a major factor in the survival, successful reproduction, and ontogenetic development of wolves [88]. Therefore, quite evidently, social embedding in a pack is the dominating factor in the welfare of individual wolves living in the wild. It is still suggested, based on the “selection for tameness” domestication hypothesis, that the strong urge of dogs to socially orient to, and cooperate with, humans [83], and even within their own species, emerged as a result of domestication [89]. However, recent experimental behavioural results from similarly raised and kept wolves and dogs have demonstrated this claim of dog social “uniqueness” via domestication to be false, suggesting that the social and cooperative orientation of dogs is of wolf heritage [61,82,83]. 

This means that, aside from adequate food and veterinary care, the major component in dog welfare is the quality of their social relationships. This certainly includes relationships with conspecifics. Most dogs enjoy playing with each other and enjoy each others’ company. However, it has been shown that free-living dog packs are more open and much less cooperative than wolf packs [61]. On the other hand, it is evident that dogs engage in quite a lot of complex and diverse cooperation under human guidance, as indicated by their positive motivation, urge and drive to fetch a ball, to find people under avalanches, to stop perpetrators (etc.), or to simply talk a walk with their owner. Hence, it seems that the social and cooperative orientation of wolves towards their conspecifics was, to some degree, re-orientated towards humans in the course of domestication. This increased importance of humans as social partners was recently substantiated by evidence that dogs, more-so than wolves, felt emotionally supported by familiar humans. In slightly stressful situations, these humans had a greater calming effect on the dogs than they did on similarly socialised wolves [90,91]. In other words, if a major factor of wellbeing of wolves is their social embedding in a pack, the same should be true for the role of human social partners in the case of dogs. This may not, however, be as hard wired as it may sound. Rather, early conditions and life experience result in individual dogs being socialised more or less closely with humans. Pet dog welfare seems most dependent on adequate relationships with their human masters. Although this may not be true for village dogs, it has been shown that their survival and reproduction is supported by their being able to establish and maintain positive relationships with people. 

However, what about street/stray dogs or village dogs who do not live in the close company of human partners and caregivers? This is the case for probably less than 50% of the world’s one billion dogs [4]. Street dogs do relate to people, and their survival and reproduction seems to be affected by this. Street dog welfare is obviously constrained by the lack of veterinary care, sub-optimal food availability, and by various threats, including traffic accidents and human prosecution. However, despite a growing body of literature on such dogs [92,93], it remains unclear whether and to what degree street dogs suffer from the lack of close social relationships with specific human partners. Much of this seems to depend on how individuals have been socialised. For example, the dogs at the “Wolf Science Center” (WSC, [94]) are socialised and worked with in the same way as the WSC wolves [61], and they also live in packs. These non-pet dogs clearly appreciate contact from and cooperation with people, but are happy to return to their packs after such interactions (personal observation). However, due to the lack of clear evidence regarding differences in welfare between pet dogs and street dogs resulting from the quantitative and qualitative differences in their social relationships with humans, this topic cannot be expanded upon here. 

### 7.2. What Kind of Relationships Support Dog Welfare? 

Under a misconception that is mainly related to an incorrect view of the social organisation of wolf packs, it was long accepted that that dogs need to be dominated, in order for them not to rebel against their human masters. However, field observations [88] have shown that wolves live in flat pack hierarchies with shared decision processes and no “Alpha” that forces their will through. Therefore, good relationships with socialised wolves, as well as with pet dogs, are not based on domination, but on cooperation from puppyhood on, which is rewarding for both sides. As a rule, pressure and punishment damage valuable long-term relationships and inhibit thinking and creative cooperation. However, whereas human-socialised wolves like to take the lead when cooperating with humans [61], this is hardly the case in dogs, who rather appreciate human leadership, in accordance with similar rules to those that apply to educating children or leading employees: good leaders solicit cooperation through positive motivation, being trustworthy, and—occasionally—by setting limits. In fact, human-socialised wolves and similarly raised and kept dogs, without too much difference, cooperate enthusiastically in subordination training sessions that rely on positive reinforcement (PR) without any domination [95].

To support dog welfare, their socio-cognitive skills should be respected, particularly during training. Although PR is highly effective in training dogs and other animals, this does not mean that PR is their only way of learning. As “do-as-I-do” experiments have shown [71], dogs are capable of forming mental representations, making judgments and basing their decisions on them. Along these lines, respectful and welfare-minded keeping would include supporting creative thinking on side of the dog. One means to this end is to use the do-as-I-do paradigm for training and for having fun together. This may also include starting clicker training during puppyhood. Wolf Science Center trainers, for example, occasionally practise a novelty game with their wolves and dogs (personal observation). The task for the animal is to offer “something new” in each round, knowing that a reward will follow a click if the trainer is satisfied. For example, a training session starts with the “show” command; the animal knows that it has to offer something and sits down—click and reward. In the second round, the animal again sits down—no click, no reward—so the trainee animal keeps on trying and circles a tree—click, reward. In this way, the game can run for several rounds, with the difference from PRT being evident: the animal thinks, creatively tries to offer behavioural variants, and is exceedingly attentive. Evidently, as part of the good leadership for which such cooperative actions constitute an important basis, such games are started and stopped by the human trainers. 

In other words, social orientation and cooperation is an important component of both dog and human welfare; hence, it is not sufficient to keep dogs well fed, receiving of veterinary care, and clean in boring settings.

### 7.3. Anthropomorphising and Mentalising: Is It OK?

The only tool humans have for relating to their environment is their typically human brain [5], which, at least in humans, and potentially also in wolves, evolved mainly in a social context over the last few million years [96]. It is no wonder that seeing the world, and particularly other animals, through a human lens [40,41] is a human universal [2,3]. Even machines such as cars are anthropomorphised, and mental features such as goodwill or spite are attributed to them. There is no reason to assume that other animals do this any differently, as Jakob von Uexküll formulated in his “Umwelt” concept [80]. Hence, as much as humans tend to anthropomorphise dogs and recognise them as being friendly, happy, wilful, purpose orientated, etc., dogs also probably “dogify” humans, although this is, of course, hard to test.

Generally, anthropomorphising other animals is considered to be a problematic [40,41], paternalizing approach to other animals. However, is this actually so? Based on the striking parallels in their social physiologies, brains, and, potentially, mindsets, anthropomorphising other animals may not be inappropriate, after all, if based on “educated empathy”. It is certainly not empathetic to simply assume neediness on the part of the other. Rather, being truly empathetic means considering the mood and situation of the other and responding appropriately—including on the basis of knowledge of the special needs of dogs. However, this is not fundamentally different from a partnership between humans. In order to function, within-species dyads, too, need affective empathy combined with rational knowledge. 

## 8. Conclusions

In line with the “Darwinian continuum”, wolves and dogs (or other amniotic animals) are socially not that different from humans, as a result of their shared social brains, physiologies and even social mindsets arising from their common phylogeny or convergent evolution. This kind of “Darwinian continuum” between species in terms of their social tools also supports recent results indicating that the social and cooperative orientation of dogs towards humans is basically of wolf heritage [97,98], albeit adapted during domestication to fit human needs. Hence, in light of current scientific evidence, mainstream philosophy has generally over-emphasised the socio-cognitive gap between humans and other animals. It follows that a phylogeny-based, between-species “common social toolbox” allows for “true” interspecific social relationships, such as, for example, that between humans and dogs. Wolves/dogs, and, above all, humans have evolved into complex socio-cognitive specialists. Therefore, living in adequate social relationships is a core component supporting the welfare, and even health, of companion animals, notably dogs, as well as of their human partners. The “shared social toolbox” also suggests that informed anthropomorphising of, and empathising with, other animals may not be as inappropriate as previously thought.

## Data Availability

Not applicable.
